# The role of syntaxins in retinal function and health

**DOI:** 10.3389/fncel.2024.1380064

**Published:** 2024-05-10

**Authors:** Lars Tebbe, Mashal Kakakhel, Muayyad R. Al-Ubaidi, Muna I. Naash

**Affiliations:** Department of Biomedical Engineering, University of Houston, Houston, TX, United States

**Keywords:** SNARE, syntaxin, retina, synapse, retinal disease

## Abstract

The soluble N-ethylmaleimide-sensitive factor (NSF) attachment protein (SNAP) receptor (SNARE) superfamily plays a pivotal role in cellular trafficking by facilitating membrane fusion events. These SNARE proteins, including syntaxins, assemble into complexes that actively facilitate specific membrane fusion events. Syntaxins, as integral components of the SNARE complex, play a crucial role in initiating and regulating these fusion activities. While specific syntaxins have been extensively studied in various cellular processes, including neurotransmitter release, autophagy and endoplasmic reticulum (ER)-to-Golgi protein transport, their roles in the retina remain less explored. This review aims to enhance our understanding of syntaxins’ functions in the retina by shedding light on how syntaxins mediate membrane fusion events unique to the retina. Additionally, we seek to establish a connection between syntaxin mutations and retinal diseases. By exploring the intricate interplay of syntaxins in retinal function and health, we aim to contribute to the broader comprehension of cellular trafficking in the context of retinal physiology and pathology.

## 1 Introduction

Syntaxins are integral components of the soluble N-ethylmaleimide-sensitive factor (NSF) attachment protein (SNAP) receptor (SNARE) superfamily of proteins (Hong and Lev, 2014). SNARE proteins, characterized by a conserved sequence of approximately 65 amino acids known as the SNARE domain (Jahn and Scheller, 2006), form complexes that play a crucial role in mediating membrane fusion events essential for various cellular trafficking processes. These trafficking events involve the fusion of the trafficking vesicles with the target membranes (Chen and Scheller, 2001; Yoon and Munson, 2018). They encompass neurotransmitter release at neuronal synapses (Jahn and Fasshauer, 2012; Südhof, 2013; Brunger et al., 2018; Stepien et al., 2019; Rizo, 2022), specialized ribbon synapses in photoreceptors and hair cells (Sterling and Matthews, 2005; Ramakrishnan et al., 2012; Hallermann and Silver, 2013), the transport of newly synthesized proteins from the (endoplasmic reticulum) ER to the Golgi apparatus (Adnan et al., 2019; Linders et al., 2019), and autophagy (Zhao and Zhang, 2019; Tian et al., 2021). The diverse array of SNAREs discovered underscores their ability to orchestrate numerous cellular processes, each requiring precise and specific regulation to maintain cell survival and function. Although the general concept of SNARE complex assembly is comparable across these processes, the specific members of the SNARE superfamily forming the complexes for distinct membrane fusion events exhibit high specialization (Hong and Lev, 2014).

The first reported SNARE complex involves syntaxin 1A (STX1A), synaptosomal-associated protein 25 (SNAP25), and vesicle-associated membrane protein 2 (VAMP2), facilitating neurotransmitter release at the neuronal synapse (Poirier et al., 1998; Rehman et al., 2014). This complex serves as an archetype for the general structure of the SNARE core complex formed in all SNARE mediated membrane fusion events. The complex is formed through the interaction of STX1A, bound to the target membrane via its transmembrane domain (t-SNARE), with SNAP25 (t-SNARE) and VAMP2 (v-SNARE) which is bound to the membrane of the merging vesicle. The interaction between these three SNAREs is mediated by STX1As SNARE domain interacting with one of the two SNARE domains of SNAP25, while VAMP’s SNARE domain interacts with the other (Rehman et al., 2014). The core of these interacting SNARE domains consists of 15 layers of hydrophobic residues, with one arginine and three glutamines in the central O-layer (Yoon and Munson, 2018). The SNARE providing the arginine is referred to as R-SNARE and the SNAREs providing the three glutamines are referred to as Q-SNARES. In the example of the neurotransmitter release complex, STX1A and SNAP25 act as Q-SNAREs, while VAMP2 serves as the R-SNARE (Rehman et al., 2014). Although in most cases Q-SNAREs are t-SNAREs and R-SNAREs are v-SNAREs, exceptions exist, such as the R-SNAREs Ykt6 and Sec22B functioning as t-SNAREs and the Q-SNAREs GS15, Bet1, and Slt1 acting as v-SNARES (Hong and Lev, 2014). A newer nomenclature further categorizes Q-SNAREs into Qa-, Qb- and Qc SNARE motifs, with STX1A as Qa-SNARE and SNAP25 as Qb- and Qc-SNARE in the case of the neurotransmitter release example (Jahn et al., 2023).

The initial complex formed by SNAREs, specifically STX1A, VAMP2 and SNAP25 in the neurotransmitter release complex, is called the trans-SNARE complex. This designation arises from the fact that it includes SNAREs localized on two membranes that have not yet merged (Yoon and Munson, 2018). The interaction within the SNARE domains in this initial complex initiates at their N-terminus and progresses toward their C-terminus through a process known as zippering (Walter et al., 2010; Südhof, 2013; Prashad and Charlton, 2014; Yu et al., 2018; Jahn et al., 2023). Zippering is crucial for overcoming repulsion between the merging membranes, allowing the approach of target and vesicle membranes, ultimately leading to their fusion. Following membrane fusion, the *cis*-SNARE complex forms, as all involved SNAREs are now localized on the same membrane (Yoon and Munson, 2018). The assembly of the *cis*-SNARE complex is followed by the formation of an initial fusion stalk, which evolves into an expanding fusion pore, enabling cargo release. The precise role of the SNARE complex in these final steps remains somewhat unclear, with evidence indicating its necessity to regulate the size and expansion of the fusion pore. However, the exact mechanism of this regulation is not fully understood (Jahn et al., 2023). Upon completion of cargo release, the SNARE complex undergoes recycling by the ATPase NSF through an indirect interaction with the *cis*-SNARE complex, mediated by SNAP adapter proteins (Söllner et al., 1993; Ryu et al., 2016; Jahn et al., 2023).

**Figure 1:**
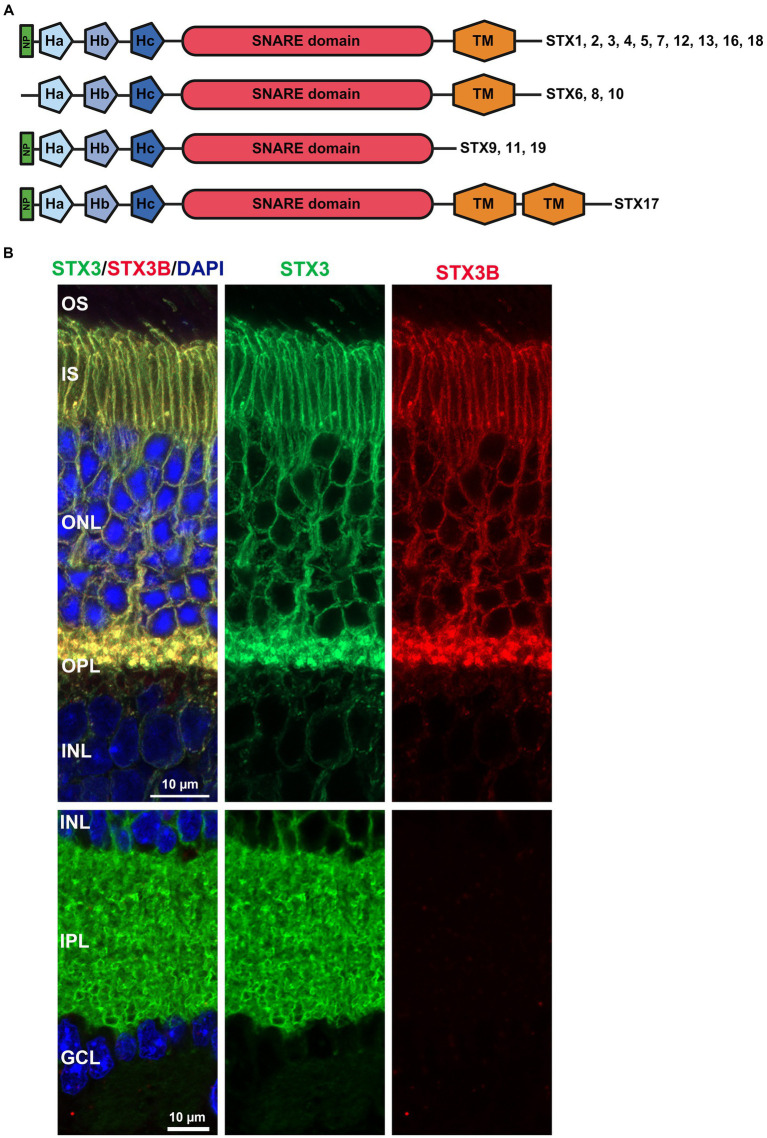
**(A)** Domain structure common to all human Syntaxins identified thus far. NP: N-terminal peptide mediating interactions between SNARE complex and SM proteins like STXBP1 and Sec1. The three helices Ha, Hb, Hc form the regulatory H_abc_ domain, while TM represents the transmembrane domain. Created with BioRender.com. **(B)** IHC with two STX antibodies (STX3 detecting all four mouse STX3 isoforms, STX3B specific for isoform B). STX3B is exclusively expressed in the photoreceptor cells.

Within the SNARE complex, syntaxins are considered particularly crucial for regulating complex formation and, consequently, initiating membrane fusion events (Rehman et al., 2014). The ability to regulate fusion is facilitated by a motif of three antiparallel helixes (H_abc_) located at the N-terminus (Dulubova et al., 1999; Dawidowski and Cafiso, 2013). This motif interacts with the syntaxin’s SNARE domain, leading to a closed conformation that prevents interaction with the SNARE domains of other complex members, thereby impeding complex assembly. While this regulatory domain is predominantly found on syntaxins (Qa-SNARE), rare examples of Qb- and Qc-SNAREs containing a H_abc_ motif also exist (Jahn et al., 2023). In addition to this regulatory motif, some syntaxins feature a short N-terminal peptide necessary for mediating interaction between the core SNARE complex and the SM proteins Sec1 and Munc18 (also known as syntaxin binding protein 1, STXBP1), essential for an efficient membrane fusion event (Rathore et al., 2010; Hong and Lev, 2014). Following the regulatory N-terminal peptide and H_abc_ motif is the SNARE domain, and at the C-terminal end, the transmembrane domain (Hong and Lev, 2014; Jahn et al., 2023) (Figure 1A). While most syntaxins share this structural composition, variations exist; for example, some syntaxins lack the transmembrane domain and require palmitoylation for membrane binding (Fukasawa et al., 2004; Hong and Lev, 2014) (Figure 1A). Conversely, syntaxin 17 (STX17) contains two transmembrane domains (Figure 1A), resulting in a hairpin formation crucial for targeting it to the autophagosome (Masuda et al., 1998; Jahn et al., 2023).

This review focuses on the role syntaxins play in the retina, emphasizing new findings in their expression patterns and interactions with retina-specific proteins. Additionally, it provides an overview of the role syntaxins in the pathogenesis of retinal diseases.

## 2 Syntaxins in the retina

**Figure 2:**
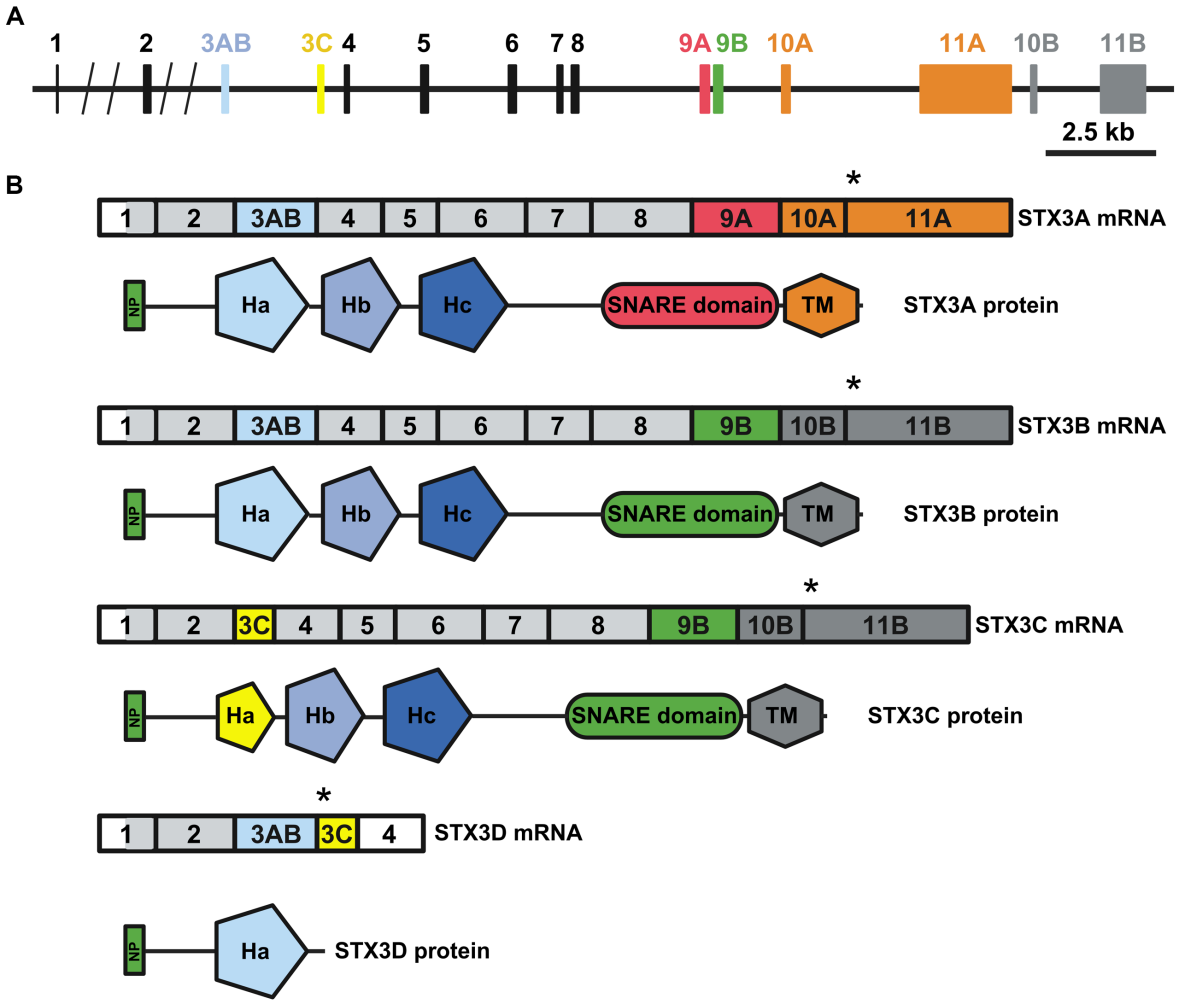
**(A)** The mouse *Stx3* gene. The different exons included in the different STX3 isoforms are highlighted with colors. **(B)** Transcript (upper) and protein (lower) resulting from differential splicing of the *Stx3* gene. Relation of exons to protein domains depicted by color choice. Asterisk highlights the stop codon, light grey region highlights start of translation. Domains: NP: N-terminal peptide; H_a_, H_b_ and H_c_: regulatory helices; SNARE: SNARE domain; TM: transmembrane domain, Designed based on Curtis et al. (2008). Created with BioRender.com.

An initial study by Sherry et al. (2006) identified four syntaxins in the mouse retina (STX1-4). While these four syntaxins adhere to the classic syntaxin structure, comprising the N-terminal peptide, the regulatory H_abc_ motif, the SNARE domain and one transmembrane domain, differences exist in specific isoforms. Both STX1 isoforms A and B exhibit high homology and are expressed in the mouse retina (Bennett et al., 1992, 1993; Ruiz-Montasell et al., 1996; Kaneko et al., 2011). However, there is no available data on retina-specific isoforms for STX2 and STX4. STX2A, B and C exhibit a broad expression pattern, while STX2D appears to be restricted to the brain (Quiñones et al., 1999). Nonetheless, a study specifically targeting the retinal expression of different STX2 isoforms is needed. For STX4, information is even scarcer, with three isoforms listed at NCBI but no study addressing exclusive function or expression pattern for these specific isoforms. While the isoforms for STX1, 2 and 4 vary only in certain parts of their amino acid sequence; the overall domain composition is unchanged (Figure 1A). The four isoforms described for STX3 (A, B, C, and D) show more structural differences (Figures 1B, 2A,B) (Curtis et al., 2008). RT-PCR based expression studies found STX3B to be the only isoform expressed in the retina, while the remaining isoforms were absent (Curtis et al., 2008). Differential splicing causes STX3A and B to contain all the motifs, but the specific sequences of the SNARE and transmembrane domains differ (Figures 2A,B). STX3C shares the transmembrane and SNARE domain with STX3B but has a unique H_a_ motif in the regulatory H_abc_ domain. Splicing together exon 3AB (H_a_ motif of STX3A and B) and exon 3C (H_a_ motif STX3C) results in frameshift creating a stop codon at the beginning of exon 3C in the isoform STX3D. Thus, STX3D lacks both a SNARE and a transmembrane domain and only contains the H_a_ motif (Curtis et al., 2008).

In addition to the four syntaxins identified Sherry et al., more syntaxin isoforms with an impact on retinal function were identified. These include STX18 (Hatsuzawa et al., 2000; Hirose et al., 2004; Aoki et al., 2008; Nishiwaki et al., 2013), which shares the normal structure, STX6 (Kubota et al., 2002) lacking the N-terminal peptide, and STX17 (Yoshii and Mizushima, 2017; Dorion et al., 2021), which includes two consecutive transmembrane domains (Figure 1A).

### 2.1 Syntaxin 1: implications for retinal function

STX1 was identified at the conventional synapses within the inner plexiform layer (IPL), where the second-order neurons connect with retinal ganglion cells (Morgans et al., 1996; Sherry et al., 2006). Positioned on the presynaptic side of these synapses, STX1 facilitates neurotransmitter release. Additionally, STX1 expression was observed in the outer plexiform layer (OPL) and the cell bodies of amacrine cells (Ruiz-Montasell et al., 1996; Sherry et al., 2006). The presence of STX1 in amacrine cell bodies prompts questions about its specific function in this context. Although potential roles, such as neuropeptide secretion and the trafficking of transporters and ion channels, have been suggested [summarized in Sherry et al. (2006)], empirical studies validating these functions are currently lacking. A knockout study targeting STX1A revealed modest structural changes in retinal layering. Specifically, an increased thickness was noted in the OPL, accompanied by a reduction in the dendrite volume of rod bipolar cells (Kaneko et al., 2011). Despite these alterations, no significant functional decline in the knockout retina was reported. This lack of functional decline may be attributed to a compensatory effect of the second STX1 isoform, isoform B (STX1B), highlighting the intricate interplay between STX1 isoforms in maintaining retinal function.

### 2.2 Syntaxin 2: expression and uncertainties in retinal function

Syntaxin 2 (STX2) exhibits predominant expression in amacrine cells, an inhibitory subset of neurons, located within the inner nuclear layer (INL) of the retina (Sherry et al., 2006). Amacrine cells are known to use GABA or glycine as their primary neurotransmitter (Wässle and Boycott, 1991; Sherry and Yazulla, 1993; Sherry et al., 2006). Co-labelling experiments, utilizing markers for GABA (glutamic acid decarboxylase) and glycine (glycine transporter 1), revealed STX2 expression in both subsets (Sherry et al., 2006). The co-localization was notably strong in the cell body and somewhat weaker in the synapses of amacrine cells within the IPL. Unlike STX1, STX2 does not appear to be localized at conventional presynapses (Sherry et al., 2006). The precise function of STX2 in the retina remains largely elusive, given the limited number of functional studies conducted to date. Despite its expression in amacrine cells, specific details regarding the functional role of STX2 in these cells and its contribution to retinal processes are yet to be explored and understood.

### 2.3 Syntaxin 3: orchestrating ribbon synapse dynamics in the outer plexiform layer

In the OPL, where photoreceptor signals interface with second order neurons, syntaxin 3 (STX3) emerges as the exclusively expressed syntaxin (Morgans et al., 1996; Sherry et al., 2006; Curtis et al., 2010). Of the four known isoforms (A, B, C, and D), STX3B specifically associates with vesicle release at the ribbon synapse. The OPL signal transduction relies on ribbon synapses, distinct from conventional synapses, exclusive to sensory cells like photoreceptor cells (rods and cones), inner and outer hair cells in the cochlea, and vestibular hair cells (Moser et al., 2020). Unlike conventional synapses facilitating all-or-nothing signal transmission, ribbon synapses enable a more graded and sustained release (Tom Dieck and Brandstätter, 2006). Facilitating this graduated release, ribbon synapses harbor a pool of glutamate-filled vesicles organized around a central structure known as the ribbon (Usukura and Yamada, 1987; Thoreson, 2021). These vesicle are categorized into a rapidly releasing pool (RRP) and a reserve pool (RP), associated with the fast, short responses and slower, sustained responses, respectively (Usukura and Yamada, 1987; Palmer, 2010; Datta et al., 2017). The SNARE complex, which facilitates the fusion of glutamate-filled vesicles at the ribbon synapse, shares the v-SNARE VAMP2 and the t-SNARE SNAP25 with the SNARE complex at conventional synapses (Morgans et al., 1996; Sherry et al., 2006; Curtis et al., 2010). However, in contrast to the involvement of t-SNARE STX1A in conventional synapses, STX3B emerges as the second t-SNARE in forming the SNARE complex at the ribbon synapses. Given the rapid release of the RRP, a priming step is necessary, where vesicles are loaded with SNARE complex components essential for subsequent membrane fusion and vesicle release (Thoreson, 2021). Remarkably, a recent study demonstrated that this vesicle priming is not exclusive to RRP vesicles but also occur to the RP vesicles (Datta et al., 2017). The precise mechanism and the specific components of the SNARE-complex at the ribbon synapse attached to vesicles in both pools during priming remain is not fully unraveled.

Evidence for the localization of STX3B at the synaptic terminals of bipolar neurons in the IPL was obtained in an initial study performed in the goldfish retina (Curtis et al., 2010). RT-PCR revealed both STX3A and STX3B expressed in the goldfish retina, with STX3B expression being seven times as high as STX3A. Immunohistochemistry utilizing an antibody targeting the N-terminus of STX3A and STX3B revealed a robust staining at both the OPL and IPL in the goldfish retina (Curtis et al., 2010). Given the dominance of STX3B expression in the goldfish retina, the bulk of the signal was assigned to STX3B. Co-labeling of STX3 and the synaptic vesicle marker SV2 in isolated bipolar neurons confirmed the localization at the synaptic terminals of goldfish bipolar neurons (Curtis et al., 2010). A follow-up study in the mouse retina using an antibody targeting the very N-terminus of mouse STX3 shared by all four isoforms confirmed this result in the mouse retina (Liu et al., 2014). Co-labelling of STX3 with Ctbp2/Ribeye in isolated bipolar cells demonstrated the localization of STX3 at the synaptic terminus of bipolar cells. Given only mRNA of STX3B could be identified in the mouse via RT-PCR, STX3 labelling in the OPL and IPL was assigned to STX3B (Curtis et al., 2008; Liu et al., 2014). However, a co-staining of retinal sections with an antibody detecting all four STX3 isoforms versus an antibody specific for STX3B (Zulliger et al., 2015) performed our lab found STX3B labelling to be absent from the IPL (Figure 1B). Thus, further studies are required to unravel the precise expression of STX3 isoforms in the different retinal layers.

An initial expression study showed that STX3 is not restricted to the OPL and IPL in the retina, but seems to be also expressed in the photoreceptor inner segment (IS) (Sherry et al., 2006). While the function of STX3B at the ribbon synapses of the OPL is well described, its role at the IS and the specific STX3 isoforms mediating this function requires further investigations. The IS serves as the hub for energy generation and protein synthesis in the photoreceptor cell, while light detection occurs in the outer segment (OS), a modified primary cilium. The OS consists of stacked discs that accommodate the essential proteins for phototransduction. To maintain the function and structure of the OS, proteins synthesized in the IS must be transported to the OS through the connecting cilium (CC), a slender bridge connecting both compartments of the photoreceptor. Efficient loading and transport of these proteins are crucial due to the continuous shedding of disc at the apical end, which are replenished with new disc at the distal end (Young, 1967; Young and Bok, 1969). This results in a daily turnover of approximately 10% of all OS proteins. STX3B was identified to interact with, among other proteins, the OS proteins peripherin 2 (PRPH2, formerly known as RDS) and rod outer segment protein 1 (ROM1), both from the tetraspanin family (Zulliger et al., 2015). PRPH2 is essential for membrane curvature and discs flattening in rods and cones (Molday et al., 1987; Arikawa et al., 1992; Boesze-Battaglia et al., 1998; Wrigley et al., 2000). Mutations in PRPH2 cause inherited retinal diseases (IRDs) leading to structural and functional decline and photoreceptor cell death [reviewed in Stuck et al. (2016) and Tebbe et al. (2020)]. ROM1, an interactor of PRPH2, plays a role in the precise sizing and alignment of discs, contributing to the structural fine-tuning of the photoreceptor OS (Clarke et al., 2000; Lewis et al., 2023).

**Figure 3:**
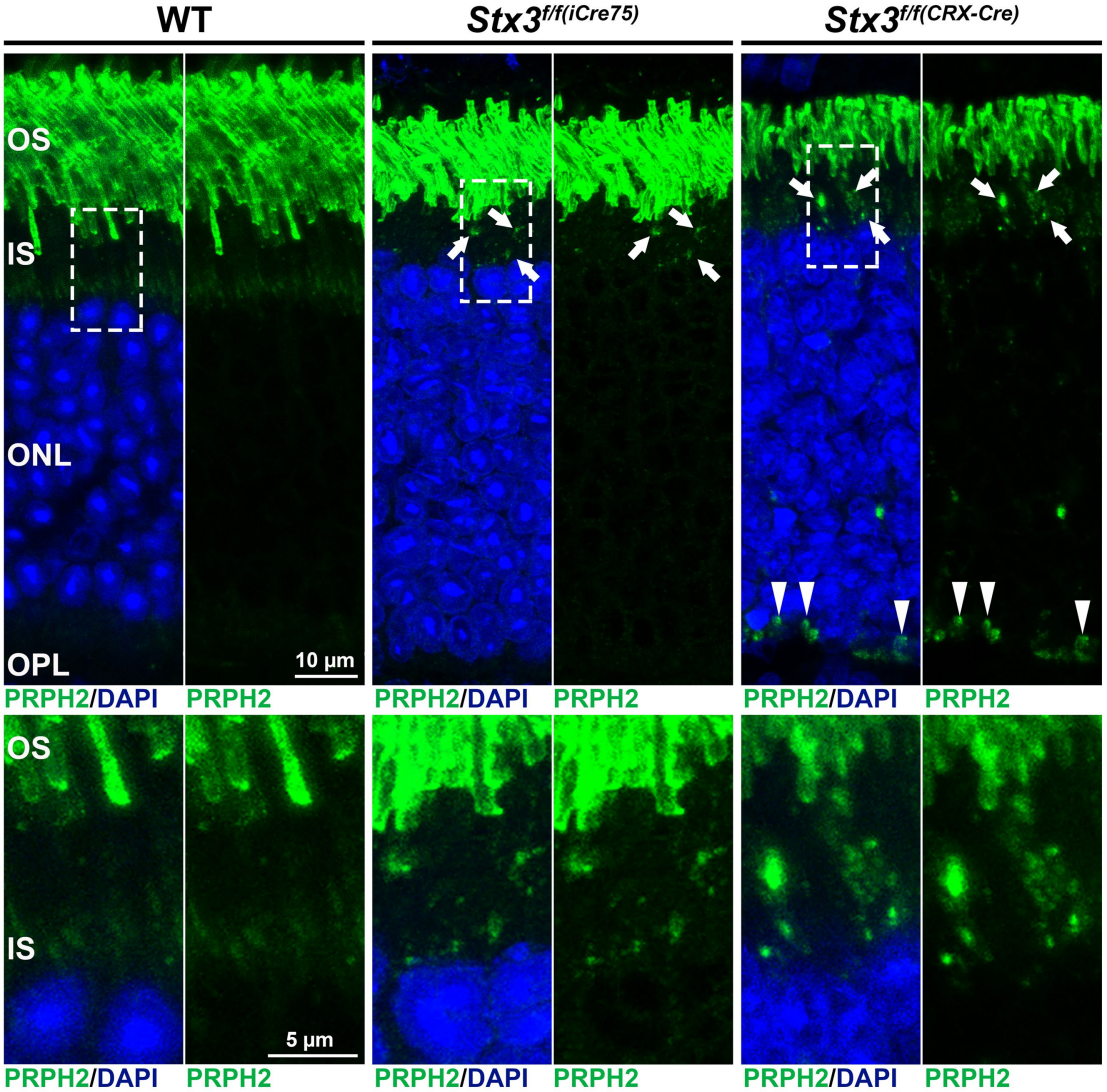
Mislocalization of PRPH2 is evident in the STX3 conditional knockouts *Stx3^f/f(iCre75)^* and *Stx3^f/f(CRX-Cre)^*. Dashed boxes in upper panels highlight the areas magnified in the lower panels. Mislocalization in IS is highlighted with arrows, while mislocalization in ONL is marked with arrowheads. OS, outer segment; IS, inner segment; ONL, outer nuclear layer; OPL, outerplexiform layer.

**Figure 4:**
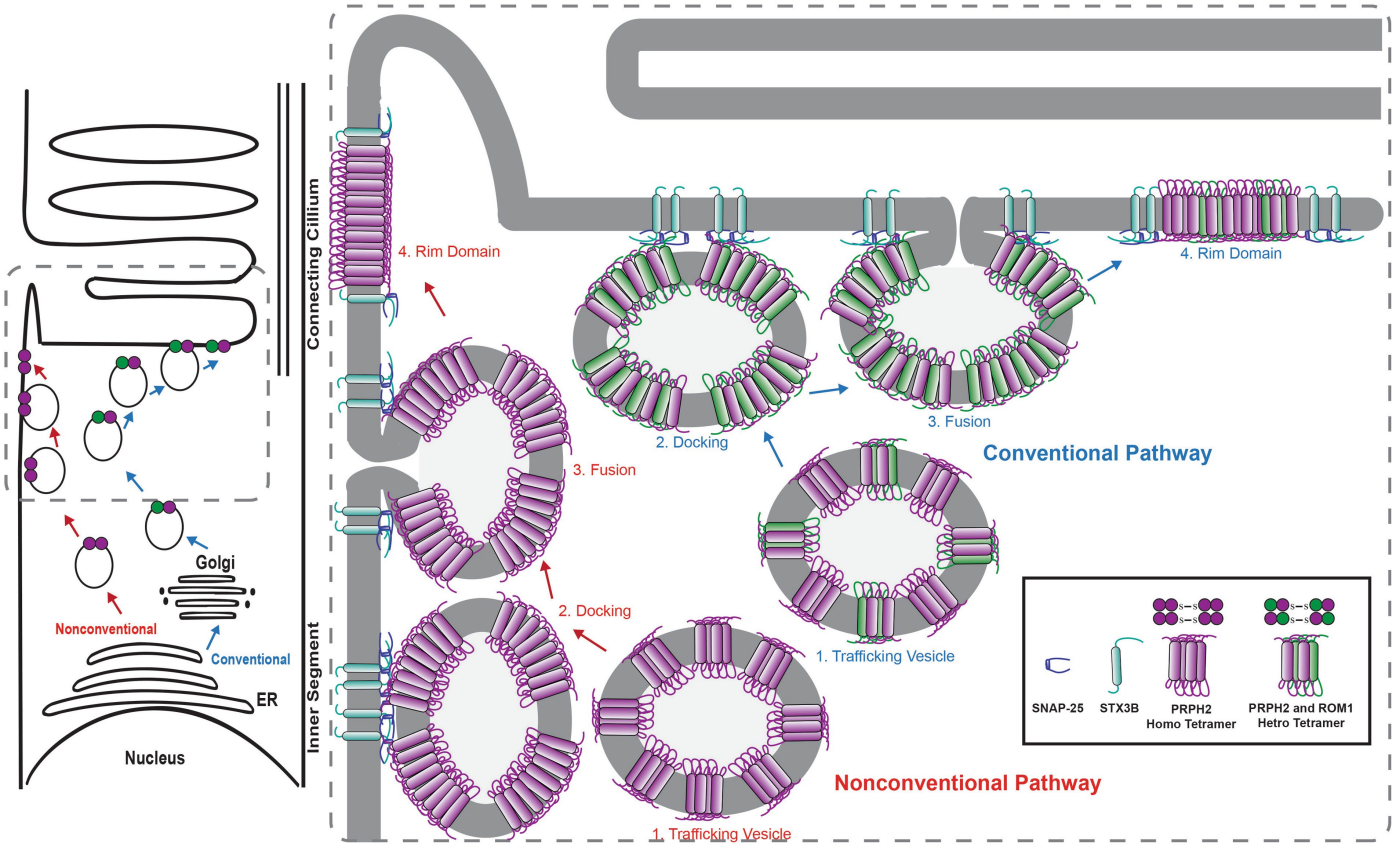
Scheme describing the hypothesized involvement of STX3B in the transport of PRPH2 and ROM1 towards the outersegment. STX3B mediates the merging of both conventional vesicles loaded with PRPH2 homotetramers and PRPH2/ROM1 heterotretramers, as well as unconvtional vesicles coming from the ER loaded exclusively with PRPH2 homotetramers with the photoreceptor membrane in the apical part of the inner segment. ER, endoplasmic reticulum.

The interactions between STX3B, which is restricted to the OPL, IPL and IS of the photoreceptor, and PRPH2 and ROM1 were surprising. Considering the complete absence of STX3B from the OS, the role of the interactions between STX3B and PRPH2/ROM1 in their trafficking from the site of synthesis towards the OS was investigated. Subsequent studies demonstrated that photoreceptor-specific conditional knockouts of STX3 (*Stx3^f/f(iCre75)^* for rod photoreceptors and *Stx3^f/f(CRX-Cre)^* for rod and cone photoreceptors) resulted in mislocalization of PRPH2, ROM1 and rhodopsin (RHO) (Kakakhel et al., 2020). PRPH2, ROM1 were found to be mislocalized in the IS as well as in the outer nuclear layer (ONL) (Figure 3). Some of these mislocalized proteins were colocalized in the IS and ONL, indicating a dependency on STX3 for their proper localization (Kakakhel et al., 2020). Interestingly, cone opsins were correctly localized in both models, suggesting a distinct trafficking mechanism for cone photopigments. Additionally, STX3B interactors, SNAP25 and STXBP1, were found aberrantly localized in the IS of the *Stx3^f/f(CRX-Cre)^* retinas. Interaction assays revealed direct associations between STX3B and PRPH2 at their SNARE and C-terminal domain, respectively (Kakakhel et al., 2020). The complex formed between the SNARE domain of STX3B and C-terminal domain of PRPH2 included known interactors such as STXBP1 and SNAP25, ROM1 and RHO. Previous studies hinted at the potential function of the C-terminus of PRPH2 as a v-SNARE (Boesze-Battaglia et al., 1998, 2007; Boesze-Battagliaa and Stefano, 2002). A proposed membrane fusion event, mediated by STX3B (t-SNARE) and the C-terminus of PRPH2 (v-SNARE) at the periciliary region of the IS, could be necessary for the trafficking of OS proteins RHO and ROM1 towards the rod OS (ROS). Furthermore, PRPH2 is known to be partially trafficked through an unconventional pathway that bypasses the trans-Golgi (Tian et al., 2014; Zulliger et al., 2015; Conley et al., 2019; Otsu et al., 2019). The glycosylation of PRPH2 can be utilized to distinguish between conventionally and unconventionally trafficked PRPH2 (Tian et al., 2014; Zulliger et al., 2015). Glycosylated PRPH2, which bypasses the trans-Golgi in an unconventional trafficking pathway is susceptible to endoglycosidase H (EndoH) treatment, while conventionally trafficked PRPH2 is resistant (Zulliger et al., 2015). A combination of an immunoprecipitation with an antibody specific to STX3B and EndoH treatment found both, conventionally and unconventionally trafficked PRPH2 to interact with STX3B (Zulliger et al., 2015). The mislocalization of ROM1, RHO (both exclusively transported conventionally), and of PRPH2 (transported conventionally and unconventionally), following knocking out STX3, together with the observation that conventionally and unconventionally trafficked PRPH2 interact with STX3B, indicate the involvement of STX3B in both transport processes (Figure 4). Yet, the precise mechanism of loading cargo towards the CC, the bridge that facilitates cargo trafficking from the IS towards the OS of photoreceptors, remains a subject of debate. Consequently, further studies are required to fully understand the role of the STX3B/PRPH2 interaction in this intricate process.

Functionally, electroretinography (ERG) demonstrated a reduction in scotopic a and b wave amplitudes by up to 55 and 66% at P30 and P60, respectively, for *Stx3^f/f(iCre75)^*. Notably, cone function remained unaffected. In comparison, *Stx3^f/f(CRX-Cre)^* mice displayed a more severe phenotype, showing no scotopic or photopic response at any investigated timepoint (P15 and P30) (Kakakhel et al., 2020). Both, scotopic a and b waves were found to be completely absent in the *Stx3^f/f(CRX-Cre)^* mice. Consistent with functional decline, both models displayed a significant decrease in ONL thickness, indicating photoreceptor death. These findings highlight that STX3 is not only crucial for vesicle fusion at the ribbon synapses but also for photoreceptor survival and proper trafficking of OS proteins. Interestingly, *Stx3^f/f(CRX-Cre)^* mice also exhibited a loss of second-order neurons, evident in decrease in INL thickness (Kakakhel et al., 2020). The occurrence of second-order neuron loss exclusively in the early-onset knockout model *Stx3^f/f(CRX-Cre)^,* and not in the later-onset *Stx3^f/f(iCre75)^* model, suggests a potential role for STX3B in the development of second-order neurons. As STX3B plays a vital role at the photoreceptor ribbon synapse, structural defects were anticipated in the STX3 knockout models (Kakakhel et al., 2020). In *Stx3^f/f(iCre75)^* mice, synaptic defects manifested as free-floating ribbons at P21 and P45 for rod and cone synapses, respectively. The *Stx3^f/f(CRX-Cre)^* model exhibited structural defects in both rod and cone synapses at P15, including free-floating ribbons. Additional defects were also observed in the *Stx3^f/f(iCre75)^* model, including vesicle accumulation, loss of arciform densities, or the complete absence of a defined ribbon structure. These defects progressed with time and were more pronounced in rod synapses, underscoring the essential role of STX3B in the development, function, and survival of the ribbon synapses.

The significance of STX3 in trafficking RHO towards the OS, as supported by the study summarized above, aligns with earlier postulations from a study conducted by Mazelova *et al* using *Rana berlandieri* retinas (Mazelova et al., 2009). In this study, STX3 was found to be enriched near the periciliary ridge, the amphibian equivalent to the mammalian periciliary membrane (Yang et al., 2010), co-localizing with its interactor SNAP25. The periciliary ridge was identified as crucial for loading cargo into the CC, an essential step in trafficking cargo towards the photoreceptor OS (Maerker et al., 2008; Yang et al., 2010). A subsequent study identified vesicle-associated membrane protein7 (VAMP7) as the v-SNARE interacting with STX3 and SNAP25 at the periciliary ridge to mediate RHO trafficking towards the OS (Kandachar et al., 2018). These studies collectively established the existence of a STX3/SNAP25/VAMP7 SNARE complex facilitating RHO trafficking to the ROS. However, findings from photoreceptor-specific knockout models of STX3 suggest a more intricate mechanism for RHO trafficking, prompting further studies to explore potential compensatory mechanisms including different retinal STX or other t-SNAREs.

### 2.4 Syntaxin 4

Syntaxin 4 (STX4) is localized in the mouse retina at horizontal cells, which are retinal second order neurons (Sherry et al., 2006). Specifically, STX4 appears to be confined to the processes of the horizontal cells in the OPL, positioned postsynaptically to the ribbon synapses at rod and cone terminals (Sherry et al., 2006; Hirano et al., 2007). Pre-embedding immuno-electron microscopy confirmed this localization, emphasizing that STX4 is absent at the ribbon synapses (Hirano et al., 2007). Co-localization of SNAP25 with STX4 at the horizontal cell processes suggest their collaboration as a SNARE complex in these cells. However, the identity of the v-SNARE in this complex remains unknown, and the precise function of the SNARE complex centered around STX4 requires further elucidation. STX4 is recognized for targeting vesicles to the plasma membrane and mediating the exocytotic release of these vesicles (Chen and Scheller, 2001; Teng et al., 2001; Salaün et al., 2004; Hirano et al., 2007). The presence of the vesicular gamma-aminobutyric acid transporter (VGAT), crucial for GABA vesicular transport, and glutamic acid decarboxylase, necessary for GABA synthesis in mammalian horizontal cells, suggests that GABA release via STX4 is probable (Haverkamp et al., 2000; Cueva et al., 2002; Jellali et al., 2002; Hirano et al., 2007; Guo et al., 2010; Lee and Brecha, 2010). Studies in mice (Sherry et al., 2006) and in rats and rabbits (Hirano et al., 2007) observed accumulations of STX4 at horizontal cell processes postsynaptic to cone pedicles, suggesting a more significant role for STX4-mediated postsynaptic vesicle fusion in cones synapses compared to rods synapses. This trend was also noted in the retinas of humans and other primates (Puller et al., 2014a,b). While initially believed to release GABA directly to cones in an inhibitory feedback mechanism (Kaneko and Tachibana, 1986; Picaud et al., 1998), subsequent studies contradicted this notion (Thoreson and Burkhardt, 1990; Verweij et al., 1996, 2003; Puller et al., 2014a). Instead, STX4 mediated GABA release from the horizontal cells appears to affect bipolar cells rather than directly impacting cone pedicles (Puller et al., 2014a). A third model for the mechanism of horizontal cell-mediated regulation of neurotransmitter release at the cone synapse was recently postulated by the Barnes lab (Grove et al., 2019). Here, GABA is released from horizontal cells into the synaptic cleft, where it binds to GABA receptors (GABAR) localized on the membrane of the horizontal cell (GABAR autoreceptors). Binding then opens the GABAR channel, which promotes the efflux of HCO_3_^−^ ions, resulting in an increase in the pH at the cleft (Grove et al., 2019). However, the impact on the pH strongly depends on the polarization status of horizontal cells. Thus, when horizontal cells are depolarized, the efflux of HCO_3_^−^ ions is not as efficient due to a reduced driving force for release (Grove et al., 2019). Additionally, depolarization drives a strong efflux of H^+^, resulting in net decrease in the pH at the synaptic cleft, which in turn then inhibits Ca^2+^ channels at the cone synapse. The reduction of Ca^2+^ influx then reduces the release of glutamate from the cone pedicles. In the case of hyperpolarized horizontal cells, the GABA mediated release of HCO_3_^−^ is increased, which results in an increase in pH at the synaptic cleft, causing an increased activity of Ca^2+^ channels and subsequently glutamate release from the cone pedicles (Grove et al., 2019). Regardless of which precise role GABA release plays, the release mechanism seems to be even more specialized in primates, with STX4 strictly localized to horizontal cells interacting with S-cones (Puller et al., 2014a,b).

Apart from its presence in horizontal cells, multiple independent studies have identified STX4 at the basolateral membrane of the retinal pigment epithelium (RPE) (Low et al., 2002; Sherry et al., 2006; Kakakhel et al., 2020). The RPE plays a crucial role in maintaining the blood-retina barrier, absorbing stray light, and recycling retinoids essential for the visual cycle (Wald and Brown, 1956; Sparrow et al., 2010; Naylor et al., 2019; Lakkaraju et al., 2020). Immunoblots using lysates of cultured RPE-J cells validated the expression of STX4 in the RPE (Low et al., 2002). Given STX4’s function in exocytosis, it is plausible that STX4-mediated vesicle fusion results in the secretion of material towards Bruch’s membrane.

### 2.5 Additional syntaxins

Beyond the previously discussed syntaxins, additional syntaxins have been found to impact retinal function and health. For instance, syntaxin 17 (STX17) plays a role in the fusion of the autophagosome with the lysosome (Yoshii and Mizushima, 2017; Dorion et al., 2021). This process involves the interplay between oxidative stress and the cluster of differentiation 36 (CD36) ligand MPE-001 (Dorion et al., 2021). MPE-001 was observed to protect cultured hTERT RPE-1 cells from oxidative stress induced by Sodiumiodate (NaIO_3_). Notably, MPL-001 restored stress responses, including an increase in STX17-positive autophagosomes (Dorion et al., 2021). While the primary effect of STX17 is in the RPE rather than the retina itself, safeguarding the RPE from oxidative damage is crucial for overall retinal health. Oxidative damage to the RPE is a hallmark feature of age-related macular degeneration (AMD), a severe retinal disease (Imamura et al., 2006; Justilien et al., 2007; Zhao et al., 2011; Datta et al., 2017; Dorion et al., 2021).

Experiments conducted on zebrafish retina uncovered the impact of the SNARE complex, comprising syntaxin 18 (STX18), BNip1, unconventional SNARE in the ER1 (USE1), and Sec22b in preventing apoptosis of maturing photoreceptor cells (Hatsuzawa et al., 2000; Hirose et al., 2004; Aoki et al., 2008; Nishiwaki et al., 2013). This complex primarily regulates retrograde vesicle transport form the Golgi-apparatus to the ER (Nakajima et al., 2004). It was demonstrated that failure to disassemble the cis-SNARE organized around STX18 led to the apoptosis of photoreceptor cells (Nishiwaki et al., 2013). Thus, the correct timely assembly and disassembly of the STX18/BNip1, USE1, Sec22b SNARE complex were found to be essential for the survival of photoreceptors.

Syntaxin 6 (STX6) was found to be upregulated in the IPL of a mouse model carrying a mutation that results in a premature stop codon in the Human retinal gene 4 (*Hrg4*) (Kobayashi et al., 2000; Kubota et al., 2002). In humans, this mutation leads to a dominant cone-rod dystrophy (Kobayashi et al., 2000). The mutant mouse model displays reduced ERG response, fundus anomalies, and retinal degeneration evidenced by thinning of the ONL (Kobayashi et al., 2000). While STX6 is typically found in macrophages, the trans-Golgi network, and early endosomes (Bock et al., 1996, 1997; West et al., 2021), its upregulation, along with STX4, in the IPL of the *Hrg4* mutant mouse has been observed. However, as of now, there is no follow-up study unravelling the precise impact of this upregulation in the disease model (Kubota et al., 2002). In addition to that, STX6 was found to regulate the transport of vascular endothelial growth factor receptor 2 (VEGFR2), a tyrosine kinase activated by binding of vascular endothelial growth factor (VEGF), from the Golgi towards the plasma membrane (Manickam et al., 2011). Proper regulation of this transport was found to be vital for the proper vascularization of the developing retina (Gaengel and Betsholtz, 2013).

## 3 Syntaxins in retinal diseases

As our understanding of the significance of syntaxins in retinal functions continues to evolve, examples linking syntaxins to retinal diseases remain limited. One such example is microvillus inclusion disease (MVID), a congenital enteropathy characterized by early-onset diarrhea, caused by mutations in myosin 5b (*MYO5B*), *STX3* or syntaxin binding protein 2 (*STXBP2*) (Aldrian et al., 2021; Al-Yaqoubi, 2023). This disease is extremely rare, with a prevalence of 1:1,000,000, even though there might be an increased prevalence in regions where consanguineous marriages are common (Akhrif et al., 2021; Al-Yaqoubi, 2023). While MVID primarily affects the gastrointestinal system, recent research uncovered its impact on retinal function (Akhrif et al., 2021; Aldrian et al., 2021; Janecke et al., 2021; Al-Yaqoubi, 2023). A study analyzing 10 MVID patients, with mutations in exons shared by both STX3 isoforms (A and B), experience early-onset severe retinal dystrophy (EOSRD) (Janecke et al., 2021). These patients exhibited visual impairments, including an inability to respond to visual stimuli and difficulties in object localization (Janecke et al., 2021). ERG testing showed a significant reduction in both rod and cone responses, indicating a progressive loss of visual function caused by STX3 mutation. A long-term impact was evident in a patient who underwent ERG testing at both one and ten years of age. The patient’s responses were diminished at one year and nearly absent at ten years of age, illustrating the progressive loss of visual responses attributed to STX3 mutations (Janecke et al., 2021). Nystagmus and anomalies in the fundus were also observed in some of these patients (Janecke et al., 2021). Interestingly, the two MVID patients with mutations exclusively in *STX3A* did not exhibit visual impairment (Janecke et al., 2021). Analyses of mRNA levels in the human retina revealed that both STX3A and B were expressed, but STX3B mRNA was significantly more abundant, potentially explaining the absence of a retinal phenotype in cases exclusive to *STX3A* mutation.

Beyond disease connections to STX3 mutations, the interaction of STX3 with PRPH2 and RHO suggest a broader impact on retinal diseases (Kakakhel et al., 2020). *PRPH2* mutations are associated with various retinal diseases (Stenson et al., 2020; Peeters et al., 2021), ranging from pattern dystrophy to macular dystrophy and retinitis pigmentosa [reviewed in Stenson et al. (2020) and Tebbe et al. (2020)]. The mislocalization of RHO, ROM1 and PRPH2 in conditional STX3 knockout models underscores the importance of the STX3 interaction in their proper trafficking (Kakakhel et al., 2020). Further exploration is needed to understand how specific disease-related mutations in RHO, PRPH2 and ROM1 may disrupt their interaction with STX3.

Mutations in STXBP1, another protein interacting with STX3, have been linked to congenital nystagmus, a symptom observed in some MVID patients (Adnan et al., 2019; Li et al., 2020; Janecke et al., 2021). A recent study discovered a new variant of congenital nystagmus within a Chinese family, attributed to a c.47A > G mutation in STXBP1, resulting in the substitution of histidine with arginine at position 16 (p.His16Arg) (Li et al., 2020). Functional tests in *C. elegans* depleted for UNC-18 (homologue to STXBP1) revealed a pronounced impairment in locomotion, which could be partially alleviated by expressing both WT and mutant p.His16Arg variant of human STXBP1. However, the rescue effect achieved with the p.His16Arg variant was notably lower (Li et al., 2020). Co-immunoprecipitation experiments uncovered that the p.His16Arg variant of STXBP1 exhibited a stronger interaction with UNC-64. Changes in the interaction of SNAREs in complexes involved in neurotransmitter release are often associated with alterations in the timing and efficiency of the neurotransmitter release, elucidating the reduced rescue capacity observed for the p.His16Arg variant of STXBP1 (Li et al., 2020). Repeating these interaction studies with Humans STX1A and STXBP1 (WT and p.His16Arg variant) revealed no change in the interaction. However, the changes in the interaction between STX3B and p.His16Arg variant were evident. Thus, the congenital nystagmus symptoms observed in the patient carrying the 47A > G mutation in STXBP1 are likely caused by a change in the interaction of the SNARE complex organized around STX3B, a complex vital for neurotransmitter release at the retinal ribbon synapse (Li et al., 2020).

STX17, involved in autophagosome fusion with the lysosome, also plays a role in maintaining retinal health (Yoshii and Mizushima, 2017; Dorion et al., 2021). While STX17 does not exclusively mediate autophagosome/lysosome fusion in the retina and RPE, there are emerging connections between STX17 and retinal diseases. Upregulation of STX17 in response to oxidative stress in cultured RPE cells suggests its potential significance in preventing AMD (Dorion et al., 2021). Additionally, dysregulation of STX17 expression could be linked to retinoblastoma formation (Huang et al., 2018), the most common malignant tumors in children (Dimaras et al., 2012; Huang et al., 2018). Using the retinoblastoma cell line Y79, it was observed that STX17 was upregulated in the context of retinoblastoma (Huang et al., 2018). Additionally, the tumor suppressor miR-124 was found to decrease the STX17 expression in the Y79 cell line. Hence, maintaining the proper expression level of STX17 and its influence on autophagy regulation appears to be a crucial factor in preventing retinoblastoma formation (Huang et al., 2018). STX17 was also found to be upregulated in primary human corneal endothelial cells depleted of *SLC4A11* via siRNA as well as in primary mouse corneal endothelial cells harvested from *Slc41a11^−/−^* mice (Zhang et al., 2020). Both are used as models for congenital hereditary endothelial dystrophy (CHED), resulting in significantly impaired vision presented as bilateral corneal edema manifesting at birth or shortly after. Both models showed a reduced levels of COX4, a mitochondrial marker, as well as reduced ATP levels. STX17, on the other hand, was found to be upregulated (Zhang et al., 2020). STX17 is known to meditate the removal of dysfunctional mitochondria via autophagy (mitophagy). Mitophagy in the CHED models is believed to be a mechanism preventing cellular stress caused by the accumulation of dysfunctional mitochondria (Zhang et al., 2020).

STX18 represents a syntaxin family member located at the ER membrane, which was found to be crucial for the correct organization of ER membranes and Golgi function (Hatsuzawa et al., 2000). The SNARE complex organized around STX18, which mediates retrograde vesicle transport from the Golgi to the ER, was found to include the neuroblastoma amplified sequence (NBAS) protein (Nakajima et al., 2004; Jahn and Scheller, 2006; Aoki et al., 2009). Mutations in *NBAS* were found to cause a variety of symptoms, including retinal dystrophy and optic atrophy (Jiang et al., 2020; Staufner et al., 2020). The precise pathomechanism of diseases related to mutations in *NBAS* is currently unknown. Experiments utilizing skin fibroblasts from patients showed impaired vesicle tethering indicating a disruption in the function of the STX18 SNARE complex (Haack et al., 2015). However, symptoms caused by *NBAS* mutations are not restricted to the retina, whereby the NBAS protein was found to be involved in a multitude of additional cellular events including nonsense-meditated decay of mRNA, making it hard to single out the precise impact of STX18 complex dysfunction and symptoms caused by *NBAS* mutations (Anastasaki et al., 2011).

The role of STX1 in retinal health is evident from the knockout study mentioned earlier (Kaneko et al., 2011). The human STX1A gene is situated on chromosome 7q11.2, and the deletion of a 1 centimorgan region on this chromosome, encompassing the STX1A gene, is associated with Williams syndrome (Nakayama et al., 1997, 1998; Botta et al., 1999), characterized by cognitive phenotypes, cardiovascular anomalies, and visual impairment (Bellugi et al., 1999; Botta et al., 1999; Castelo-Branco et al., 2007). Structurally, the retina in this syndrome exhibits concave discs and an overall reduction in thickness (Castelo-Branco et al., 2007). In the STX1A knockout mouse, structural anomalies include an increased thickness of the OPL and a decreased number of dendrites in rod bipolar cells, while no significant deterioration in visual discrimination tasks indicates that retinal function remains largely unaffected (Fujiwara et al., 2006, 2007; Kaneko et al., 2011). The absence of a functional phenotype may be attributed to a potential compensation of STX1B in the STX1A knockout scenario. Since the deleted region on chromosome 7q11.2 in Williams syndrome patients encompasses more genes than just STX1A, reproducing the visual phenotype in a mouse model might require the deletion of additional genes.

## 4 Conclusion

Syntaxins, pivotal regulators involved in orchestrating membrane fusion events, are gaining prominence in the realm of retinal health and function. This review underscores the specific roles of various syntaxins within the retina. Currently, STX3 emerges as a crucial player essential for retinal function, as evidenced by knockout models showcasing a decline in retinal function and degeneration (Kakakhel et al., 2020). Beyond its involvement in neurotransmitter release at the ribbon synapse, STX3 also plays a pivotal role in the accurate trafficking of OS proteins, including PRPH2, RHO and ROM-1, as corroborated by multiple independent studies (Mazelova et al., 2009; Kandachar et al., 2018; Kakakhel et al., 2020). Notably, STX3 stands as the sole syntaxin linked to retinopathy-causing mutations, evident in the retinal phenotype observed in MVID patients carrying STX3 mutation (Janecke et al., 2021).

Despite the predominant focus of studies investigating syntaxins in the retina on unraveling the retinal function of STX3, it is becoming increasingly evident that other syntaxins play crucial roles in retinal health and function. An illustrative example is the upregulation of STX17 expression in RPE cells exposed to oxidative stress, in corneal endothelial cell lines used as model for CHED and in retinoblastoma cell lines. This underscores the relevance of other syntaxins in maintaining retinal health. Furthermore, the list of syntaxins pertinent to the retina continues to expand. Consequently, it is anticipated that ongoing studies will uncover changes in syntaxin expression in retinal disease models, further contributing to the growing list of syntaxins implicated in retinal context.

Research into the retinal function of syntaxins suggests potential differences in their roles in rod and cone photoreceptors. For instance the *Stx3^f/f(CRX-Cre)^* model, wherein STX3 is knocked out in both rods and cones, exhibits mislocalization of OS proteins in rod photoreceptor cells, while the localization of OS proteins in cone photoreceptors remains unaffected (Kakakhel et al., 2020). This implies that STX3 is crucial for OS protein trafficking in rods but dispensable for trafficking in cones. Conversely, the example of STX4 suggests the prospect of certain syntaxin being specific to either rods or cones. As previously mentioned, STX4 is highly enriched in the processes of horizontal cells postsynaptic to cone pedicels in the retinas of mice, rats and rabbits (Sherry et al., 2006; Hirano et al., 2007). In humans and other primates, STX4 appears to be even more restricted, being found only in the horizontal cells processes postsynaptic to S-cones (Puller et al., 2014a,b). The exploration of rod- and cone-specific syntaxins and SNARE complexes, along with understanding their impact on diseases related to rods and cones, presents an exciting avenue for further studies.

## Author contributions

LT: Conceptualization, Writing – original draft. MK: Writing- original draft, Visualization, Data curation, Investigation. MA-U: Funding acquisition, Supervision, Writing – review & editing. MN: Funding acquisition, Supervision, Writing – review & editing.
